# Everybody's Page

**Published:** 1891-03-21

**Authors:** 


### Everybody's Page.

It is a great blow to fair householders that the Royal Commission on the relations existing between employers and employed is not to include those between mistresses and servants.

---

A funeral reform association is evidently wanted on the Gold Coast, where burial expenses are so heavy that they mean almost ruin to the unfortunate peasant. If he lose wife, child, father, or, most important of all, uncle, custom, that most tyrannical of rulers, requires him to sacrifice goats at the funeral, and subsequently at stated intervals to fire off guns, provide rum for all comers for a week after death (a period when, doubtless, by a curious coincidence, there is a great thirst in the land), and to repeat the process after an interval of six weeks, and again after a year has elapsed. In order to pay for all this outlay he has to borrow money for which interest of from fifty to a hundred per cent. has to be given. Until the principal is repaid he is a slave, and has to work two days in each week for the lender.

London is said to require a daily supply of more than 150 million gallons of water. Of this 15 million gallons come from deep wells in the chalk, the rest from other sources, principally from the rivers Thames and Lea. The water from the chalk, though very free from organic impurities, is exceedingly hard, and if not softened costs the householder endless expense for cleaning boilers and obstructed pipes.

---

Children possess the qualities of imagination and originality in a greater degree even than novelists. An example of the truth of this was presented to us by a youthful friend a few days ago. In the course of a lesson on geography the form of youthful aspirants after the tree of knowledge was asked what Yarmouth is famous for. "Herrings and bloaters," was the prompt and correct reply received. Our friend in his turn was then questioned as to the difference between a herring and a bloater. He replied with a promptitude worthy of a nearer approach to the truth, "One is the cock and the other the hen!"

